# Enhancing cardiovascular artificial intelligence (AI) research in the Netherlands: CVON-AI consortium

**DOI:** 10.1007/s12471-019-1281-y

**Published:** 2019-05-20

**Authors:** J. W. Benjamins, K. van Leeuwen, L. Hofstra, M. Rienstra, Y. Appelman, W. Nijhof, B. Verlaat, I. Everts, H. M. den Ruijter, I. Isgum, T. Leiner, R. Vliegenthart, F. W. Asselbergs, L. E. Juarez-Orozco, P. van der Harst

**Affiliations:** 1 University of Groningen, University Medical Center Groningen, Department of Cardiology, Groningen, The Netherlands; 2 Go Data Driven, Amsterdam, The Netherlands; 3 Cardiologie Centra Nederland B.V., Utrecht, The Netherlands; 4 Department of Cardiology, Amsterdam Universities Medical Centre, location VU Medical Centre, Amsterdam, The Netherlands; 5 Siemens Healthcare Nederland B.V., Den Haag, The Netherlands; 6 Binx.io B.V., Amsterdam, The Netherlands; 7 Department of Cardiology, Division Heart and Lungs, University Medical Center Utrecht, University of Utrecht, Utrecht, The Netherlands; 8 University of Groningen, University Medical Center Groningen, Department of Radiology, Groningen, The Netherlands; 9 Durrer Center for Cardiovascular Research, Netherlands Heart Institute, Utrecht, The Netherlands; 10 Institute of Cardiovascular Science, Faculty of Population Health Sciences, University College London, London, UK; 11 Institute of Health Informatics, University College London, London, UK; 12 Turku PET Centre, Turku University Hospital and University of Turku, Turku, Finland; 13 University of Groningen, University Medical Center Groningen, Department of Genetics, Groningen, The Netherlands

**Keywords:** Machine learning, Artificial intelligence, Cardiovascular disease, CVON-AI consortium

## Abstract

**Background:**

Machine learning (ML) allows the exploration and progressive improvement of very complex high-dimensional data patterns that can be utilised to optimise specific classification and prediction tasks, outperforming traditional statistical approaches. An enormous acceleration of ready-to-use tools and artificial intelligence (AI) applications, shaped by the emergence, refinement, and application of powerful ML algorithms in several areas of knowledge, is ongoing. Although such progress has begun to permeate the medical sciences and clinical medicine, implementation in cardiovascular medicine and research is still in its infancy.

**Objectives:**

To lay out the theoretical framework, purpose, and structure of a novel AI consortium.

**Methods:**

We have established a new Dutch research consortium, the CVON-AI, supported by the Netherlands Heart Foundation, to catalyse and facilitate the development and utilisation of AI solutions for existing and emerging cardiovascular research initiatives and to raise AI awareness in the cardiovascular research community. CVON-AI will connect to previously established CVON consortia and apply a cloud-based AI platform to supplement their planned traditional data-analysis approach.

**Results:**

A pilot experiment on the CVON-AI cloud was conducted using cardiac magnetic resonance data. It demonstrated the feasibility of the platform and documented excellent correlation between AI-generated ventricular function estimates as compared to expert manual annotations. The resulting AI solution was then integrated in a web application.

**Conclusion:**

CVON-AI is a new consortium meant to facilitate the implementation and raise awareness of AI in cardiovascular research in the Netherlands. CVON-AI will create an accessible cloud-based platform for cardiovascular researchers, demonstrate the clinical applicability of AI, optimise the analytical methodology of other ongoing CVON consortia, and promote AI awareness through education and training.

## What’s new?

- Artificial intelligence (AI) is a promising method for analysis and interpretation of complex data.
- In contrast to other fields, little AI can be found in the cardiovascular research arena in the Netherlands.
- The authors feel there is an urgent need to catalyse the update of AI in the cardiovascular research arena in the Netherlands.
- The CVON-AI consortium aims to create an easily accessible cloud-based platform for intuitive AI implementation in a wide spectrum of cardiovascular datasets.
- Our goal is to demonstrate the clinical applicability and value of AI in cardiovascular disease, and to extend the analytical methodology of ongoing Dutch cardiovascular research consortia.

## Background

### Cardiovascular disease and research in the Netherlands

Cardiovascular disease still represents a major cause of morbidity and mortality in Europe [1]. Although it has become the second largest cause of mortality in the Netherlands, it has been estimated that cardiovascular disease burden will increase by 50% within the next 25 years [2]. The profile of cardiovascular disease includes acute (coronary syndromes) and chronic conditions (heart failure, valvulopathies, and atrial fibrillation) for which diagnostic and therapeutic improvements have been achieved in the last few decades. Nevertheless, the nature of cardiovascular disease is that of a multifactorial system that encompasses a complex bus of genetic, biological, environmental, and lifestyle determinants. This complexity is also visible in the variability of the disease expression itself, which constitutes an added challenge for our efforts to mitigate its advance.

Great efforts have been directed towards the extensive characterisation of cardiovascular risk factors, as they hold promise in the generation of mechanistic models that allow for better prevention, detection, and treatment of cardiovascular disease in both men and women. To address the increasing burden of cardiovascular disease, the Dutch Heart Foundation has prioritised three main areas of interest since 2014 [3], specifically: ‘early recognition of cardiovascular disease’, ‘cardiovascular disease in women’, and ‘improved treatment of heart failure and arrhythmia’. Consequently, a number of experimental and clinical initiatives, in the form of research consortia, have been established. Such consortia have collected large amounts of patient data that harbour enormous potential to enlarge our knowledge on the mechanistic pathways in cardiovascular disease in order to improve early recognition, facilitate proper clinical decision-making, and generate personalised therapeutics. However, the planned and performed methodological approaches within these consortia mainly rely on traditional statistical analyses, which may be insufficient to discover more complex relations within the data that may be relevant in the study of cardiovascular disease.

### Artificial intelligence and machine learning

Artificial intelligence (AI) is defined as ‘the theory and development of computer systems that are able to perform complex tasks requiring human level intelligence’ [4]. Although several other definitions are used, all propose the use of computer-based models that can perform specific activities with a level of complexity at least comparable to (and likely beyond) that of human level intelligence. Machine learning (ML), on the other hand, represents the family of algorithms able to elucidate and implement (i.e. learn and apply) complex patterns from available data through iterative optimisation processes to perform prediction or classification tasks in new data [5]. The niche of ML is located at the intersection of computer science, statistics, and subject-matter expertise [6]. A key feature of machine learning is its capability to explore large amounts of data for very high-dimensional non-linear interactions which would likely be unattainable through traditional modelling [7]. The concepts of AI and ML have inaccurately been used as synonyms. However, it is relevant to know that AI rather relates to the application of task optimisation systems while ML describes the methods or algorithms that underpin such applications.

Historically, an initial idea of programming human level intelligence into machines aroused interest during the 1950s and 1960s. Later on, a second AI development wave was initiated during the 1990s with the incorporation of various ML algorithms that could elucidate (i.e. learn) dependencies between structured data and outcomes of interest. Despite the value of these models and the emerging knowledge about data inference, ML only flourished to a limited extent, mainly due to limited computational power, limited size of available datasets, and the need for expert-designed features. Nowadays, unrestrained scalability of data storage and availability of novel graphics processing units (GPUs) have turned ML theories into useful AI applications. Moreover, these factors have allowed the emergence of a novel iteration of a particular set of ML algorithms called neural networks. Such refinement (i.e. deep neural networks) has delivered complex and powerful algorithms that are remarkably effective in image processing and are currently conceptualised as deep learning.

This phenomenon has permeated several domains of scientific knowledge with immediate extension to key performance areas in industry (e.g. Google’s search engine, e‑mail spam filters, game mastering by Alpha Zero [8], and driverless cars by Tesla). Notably, the open-source character of the field has caused further acceleration with free software tools and libraries, as well as the publication of many of the codebases [9–11]. At the same time, implementation of AI in medical sciences has begun and is quickly gaining pace [12, 13]. For instance, in April 2018 the first AI-based automatic medical tool was cleared by the FDA for the detection of diabetic retinopathy [14]. It is clear, therefore, that promoting AI applicability in cardiovascular medicine is warranted.

### The cloud

In computing, the cloud is defined as ‘a network of remote servers hosted on the Internet and used to store, manage, and process data instead of local servers or personal computers’ [15]. After initially investing primarily in storage services, major companies in the field of computing have shifted their focus to providing customers with flexible, highly scalable platforms to run their applications on. Integration of, and communication between, these systems is supplied and managed by cloud computing providers, without the need for the user to be aware of the hardware configuration. Cloud systems adapt instantaneously to the customers’ changing demands, and most of them work on a pay-per-use basis. The on-demand availability of these systems saves customers’ start-up investments and releases them from the need to have expertise and hire personnel to maintain large-scale computing systems. Consequently, the cloud provides researchers with a technical platform that allows them to develop small-scale, low-budget pilot or try-out studies that can be performed on a relatively small number of computers, and that can later scale up into promising full projects.

### Applicability of AI in cardiovascular research

Three elements are necessary for the successful implementation of machine learning-based AI, namely: sufficiently large amounts of data, large computational power provided by GPUs, and a sufficiently complex problem. The last-mentioned is necessary because it is likely that simpler problems may be adequately and robustly modelled through traditional statistical approaches [16]. The current standard in the Netherlands for these elements has been achieved through large datasets emerging from ongoing Dutch Cardiovascular Research (CVON) consortia, national high-performance computing clusters, and cloud-based computing services, as well as the recognition of the complexity of both the pathophysiological and clinical spectrum of cardiovascular disease.

Considering the current state of development in ML algorithms, the availability of cloud infrastructures and the advancement of AI into medical sciences, we have initiated a novel CVON consortium, the CVON-AI. Hence, the present report aims to outline its purposes and envisioned structure, as well as to report on the preliminary results that it is delivering.

## Methods

### The CVON-AI concept

**Fig. 1:**
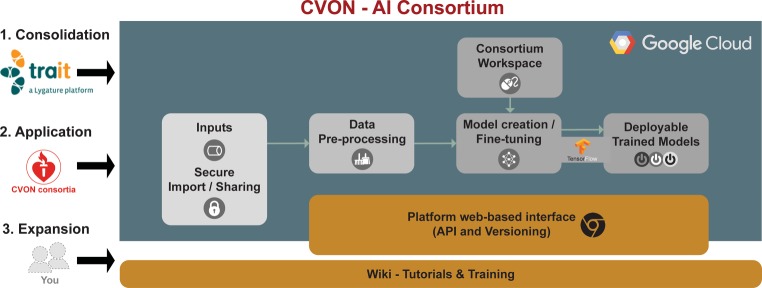
Cloud platform architecture

The CVON-AI consortium has been designed to catalyse the implementation of AI into cardiovascular disease research through: the application of ML algorithms including deep learning in large and diverse datasets, their further optimisation, the facilitation of accessibility to these methods to enhance large-scale collaborative research, and the promotion of AI awareness in the Netherlands. Figure 1 depicts the general architecture of the CVON-AI consortium.

The CVON-AI consortium will initially demonstrate the value of AI implementations in comparison with the planned standard statistical methodologies in already existing cardiovascular research scenarios (Consolidation). It ultimately strives to integrate a secure and accessible platform that allows for the utilisation of AI methodology in CVON initiatives and other available and future data-generating initiatives (Application). Thereon, the adequacy and magnitude of contribution of AI integration into clinical decision-making will be evaluated as a direct application use case through the integration of CVON-AI-generated ML models into clinical decision support tools that will be validated in randomised studies. These proceedings will be undertaken along with parallel and sustained efforts to introduce, promote, train, and constantly update (potential) users, collaborators, and other interested parties in general (Expansion). The CVON-AI consortium will be open to other investigators joining and using the developed platform and techniques at minimal costs.

### A cloud-based platform

A cloud-based platform will be developed to allow the generation of a web interface (with an open, standardised application programming interface (commonly known as API)) consisting of several building blocks to execute stepwise processes such as: secure importing of (different types of) study data, optional sharing of study data, standard and customisable preprocessing actions on study data, utilisation of standard preinstalled and customisable ML (in general, and deep learning in particular) frameworks, accessing consortium/project-specific workspaces, training of AI solutions, and their deployment with real-time interaction via the web.

The platform will deliver a standard set of untrained and pretrained models that will be made available to participating consortia over the course of time. The consortia will keep track of, and publish an index of, different versions of existing models that will be made available through the platform, and that will also remain available to the consortia’s projects after improved versions have been released. Additionally, each consortium will have its own projects and models, trained for a specific purpose, based on a corresponding dataset. Different approaches towards model architecture and parameter tuning, as well as the resulting output, may be retained in a project’s workspace, and physically persisted to cloud storage, to be selected and reviewed afterwards, at any given time.

### Data quality and safety

The CVON-AI platform will promote the reuse and combination of datasets that are uploaded to the platform. Data will be verified for aspects such as completeness, anonymisation, consistency of structure, and reliability. Especially for the purpose of combining datasets, compatibility and mapping of data structures will be central features that will be deeply embedded in the data collection components of the platform.

CVON-AI will make use of cloud technology made available by leading technology companies for the creation of an accessible platform. Consequently, platform security will rely on technology that utilises proven measures of authentication, authorisations, backup/recovery, and secure data storage. Although the data that will be processed on the CVON-AI platform will be de-identified, CVON-AI will verify the cloud technology’s compliance with data protection regulations and guidelines.

### Pilot experiment on the CVON-AI cloud platform

**Fig. 2:**
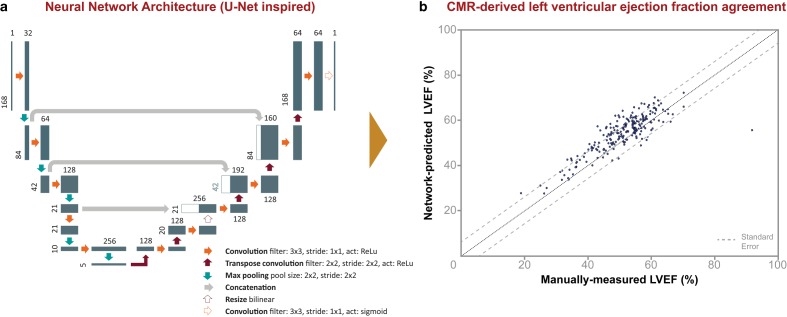
**a** Implemented U‑Net inspired neural network architecture. For each layer of the network, the height of a block represents width and height in pixels of the input and output images, and the size of data in memory in each of the model’s layers. The width of each block represents the depth, or the number of parallel filters through which data pass in each layer of the network. *Act* type of activation function; *ReLu* rectified linear unit, or rectifier, an activation that outputs zero for inputs less than zero and outputs that equal their input for inputs greater than or equal to zero. **b** Left ventricular ejection fraction (*LVEF*) determined from the contours predicted by the U‑Net model compared to the LVEF derived from manual annotations

As an exploratory step to evaluate and to showcase the feasibility and workflow of the CVON-AI towards the development of a platform for cardiovascular research, a pilot project was undertaken to navigate through the Google Cloud Platform’s technical horizon with a practical problem in hand. The objective was to train and test a deep learning model (Fig. 2a) in the cloud to perform an automated tracing of the left ventricular cavity (LVC), the myocardium, and right ventricular cavity (RVC) on short axis views from cine cardiac magnetic resonance (CMR) images in order to obtain application-based interactive estimates of cavity volumes and ejection fraction as widely used cardiac function measures.

### Raising AI awareness

Dissemination constitutes an essential objective of the CVON-AI consortium. Promotion is expected to take place via website access, presentations, road-show events, as well as education and training workshops. Involvement of cardiovascular scientists will be boosted through the involvement of companies that directly benefit from the project and of program leaders and Young-Talent-Program principal investigators from existing CVON consortia.

## Preliminary results

### Pilot experiment and platform implementation

Images from 222 patients involved in the GIPS-III study [17] were used in this pilot project. Manual annotations of end-diastolic and end-systolic frames were performed by imaging core laboratory technicians as reference for evaluation of the model. The data were uploaded to the Google Cloud Platform Storage and the code base was imported from a central source-code management system. Subsequently, a deep learning model was trained and tested (see Box 1). Notably, the utilisation of the Google Cloud Platform enabled faster training, testing and prediction, as more computational resources can be reserved for particular use from the Google servers on demand. The results demonstrated excellent correlation between AI-generated ventricular function estimates and the expert manual annotations as shown on the right side of Fig. 2.

#### Box 1 The CVON-AI pilot: technical implementation

- The trained model was an implemented variation of the U‑Net first published by Ronneberger et al. [18], which has been shown to be a well-performing model for biomedical image segmentation.
- The code base was locally developed in Python with Keras (Tensorflow backend) and versioned with a GitHub hosted git repository.
- From the source images, a random selection of 80% of the patients was taken to train the model; the other 20% was used to test the model.
- Sixteen central processing units and a single Nvidia Tesla P100 graphics processing unit were recruited and training duration was minimised to a scale of minutes (in contrast to hours).
- The program then automatically specified the appropriate images to be used for analysis (using several machine learning models). On the selected images annotations are calculated using the trained U‑Net.
- Areas are calculated from the segmented surfaces for all short axis discs of all frames that have been recorded in the patient’s study, taking into account the image resolution (cm/pixel), which can be extracted from the DICOM images’ metadata. Volumes of the different structures were calculated per frame by adding frustum volumes of the stacked areas (Eq. 1),

1 $$V=\sum _{i=0}^{n}\frac{h}{3} (A_{i}+ \sqrt{A_{i}\mathrm{*}A_{i+1}}+ A_{i+1})$$

- with *A*_*i*_ being the ordered areas of the segmented slices and *n* =number of slices −1.
- The frame with the largest volume is chosen to be end diastole, and from this frame the end-diastolic volume is selected. Likewise, the frame with the smallest volume is used for the selection of the end-systolic volume.
- The hard dice index was utilised as the main evaluation metric (Eq. 2) and was calculated for each structure and for train and test set separately.
  2 $$\text{dice index}=\frac{2\cdot \mathrm{TP}}{\mathrm{TP}+\mathrm{FP}+\mathrm{FN}}= 2 \frac{y_{\text{true}}\bigcap y_{\text{pred}}}{y_{\text{true}}+y_{\text{pred}}}$$
  with TP (true positive) being the surface made up by predicted white pixels that match white pixels in the ground truth image, FP (false positive) being the surface of predicted white pixels that do not have matching white pixels in the ground truth, and FN (false negative) the surface of predicted black pixels that do not have matching black pixels in the ground truth.

**Fig. 3:**
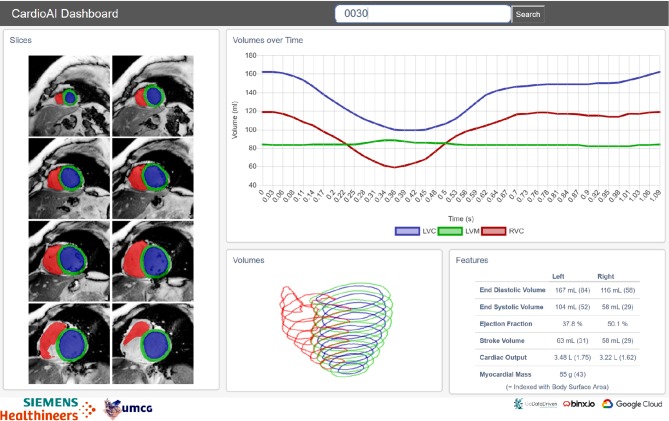
Model-backed demo web application with segmentations on a limited set of magnetic resonance images from the GIPS-III study. *LVC* volume of the left ventricular cavity, *RVC* volume of the right ventricular cavity, *LVM* mass of the left ventricular tissue, calculated from the left ventricular contours at each consecutive frame

The finalised model was then integrated into a web application (see Fig. 3) with the help of the Google Cloud Platform App Engine. The application was ultimately able to receive a CMR of a single new patient (i.e. not yet seen by the system) and to directly generate the proper segmentations of the LVC, RVC, and myocardium along with an output of estimated volumes and ejection fractions. Additionally, radial strain was calculated for the myocardium and visualised in colour for demonstration of asymmetry in the heart dynamics.

This successful implementation in the pilot study demonstrated the feasibility of the workflow intended by the CVON-AI consortium. This approach exemplified how CVON-AI will undertake training, validation, and fine-tuning of tailored ML models to analyse incoming showcase data from collaborating CVON consortia (CVON EARLY-SYNERGY and CVON RACE-V, but open for additional collaborations). This will allow timely comparison of the achieved AI results to those obtained through the analysis stated in their original research plan. It is expected that analyses undertaken through the CVON-AI initiative will render an advantage beyond traditional approaches, and therefore better exploit the potential of the large-scale data provided by these consortia.

### AI awareness

As an initial step in the raising of AI awareness through the CVON-AI consortium, GoDataDriven (a constitutional partner of CVON-AI) supported the organisation of a ‘challenge’ in the Dutch Data Science Week (visit: https://www.youtube.com/watch?v=5rW7EchLK0Y), which achieved substantial involvement in an innovative environment.

#### Upcoming challenges

Although AI has received positive responses from professionals and consumers in other areas of everyday life, acceptance of AI solutions in healthcare will surely need a wider timeframe for robust implementation considering healthcare providers, patients, and governing bodies that oversee regulations. Automating diagnostics and decision-taking on patient treatments is a delicate matter that should be addressed comprehensively. Approval and validation of developed models will be of even greater importance, compared to the governance of release trajectories of classical software solutions. Additionally, quality assurance of developed models can be precarious, since both training and validation rely on ground truth data that has been created by human observers, and must be considered potentially biased. However, if these aspects are taken care of accurately, and in detail, the provided techniques can eventually show their true potential, by improving patient care, modifying disease outcomes, and providing higher productivity at a lower cost.

## Conclusion

CVON-AI is a new consortium meant to facilitate the implementation and raise awareness of AI in cardiovascular research in the Netherlands. It is emerging at a time of rapid developments in ML algorithms that have boosted the suitability of AI solutions in many areas of knowledge. CVON-AI will create an accessible cloud-based platform for cardiovascular researchers, demonstrate the clinical applicability of AI, optimise the analytical methodology of other ongoing CVON consortia, and promote AI awareness through education and training.
